# The consistency fallacy and failures of theory embellishment

**DOI:** 10.3389/fpsyg.2013.00965

**Published:** 2013-12-25

**Authors:** Christopher H. Chatham

**Affiliations:** Department of Cognitive, Linguistic, and Psychological Sciences, Brown UniversityProvidence, RI, USA

**Keywords:** model comparison, consistency fallacy, accounting for data, Bayes Theorem, null prediction

Trafimow (2013) rejects the notion that data can militate against a theory if the data is wholly irrelevant to the theory. This very reasonable-sounding argument may not hold in the important (and common) case where competing models are being compared. The underlying issues may be clarified by way of example.

Consider the active debate on the contributions of fMRI to cognitive theory (e.g., Mather et al., 2013). It has been argued that fMRI adds little to cognitive theorizing, because data from fMRI are fundamentally irrelevant to cognitive theories pitched at the traditional computational level of analysis (Marr, 1982). A so-called “consistency fallacy” is said to be committed when data is treated as informative simply because some theory is consistent with it (Loosemore and Harley, 2010; Coltheart, 2013). This fallacy putatively arises because no other result could possibly obtain; neuroscientific data is simply irrelevant to cognitive theories at this level of analysis.

Both this argument and Trafimow's (2013) more general variant of it are, however, incorrect when any competing theory assigns a differential prior to the obtained result. Take the general case described by Trafimow (2013): some theory assigns equal likelihood to all possible outcomes of an experiment. (This is just another way of saying the data are irrelevant to it.) The observed data will nonetheless reduce our belief in the original theory as compared to some competing theory, all else being equal, if this competitor had assigned a higher prior to the obtained outcome. Conversely, the observed data will increase our belief in the original theory as compared to a competing theory, if this competitor had assigned a lower prior to the obtained outcome. This argument holds regardless of whether the observed outcome is a significant difference or a null effect; Bayes' rule of course applies in both these conditions.

That said, it is true that no evidence can be offered regarding existing theories if they all assign equal likelihood to all outcomes, or if they cannot be subject to model comparison. In practice, such a situation might less often reflect sloppy inference than a failure to adequately articulate, develop or embellish a theory.
